# Time to antibiotic administration in intra-amniotic infections: impact of cefoxitin monotherapy versus traditional antibiotic therapy

**DOI:** 10.1017/ash.2026.10748

**Published:** 2026-07-20

**Authors:** Hannah Bischoff, Sarah Tennant Withers, Caroline Jozefczyk, Joseph Kohn, R. Jake Crocker, Jasmine Lewis, Carolyn Ellison, Joseph A. Ewing, Pamela Bailey

**Affiliations:** 1 UH Rainbow Babies & Children’s: UH Rainbow Babies and Children’s Hospital, USA; 2 Pharmacy, Prisma Health Upstate, Greenville, SC, USA; 3 Prisma Health Midlands, Columbia, SC, USA; 4 University of South Carolina School of Medicine, Columbia, SC, USA

## Abstract

**Objective:**

This study addresses treatment of intra-amniotic infection (IAI) by comparing timely receipt of recommended antibiotic administration between cefoxitin and traditional antimicrobial therapy, ampicillin and gentamicin, with or without clindamycin.

**Methods:**

This retrospective cohort study was conducted at a large, multi-site health system. The combination therapy group treated with ampicillin plus gentamicin, with or without clindamycin from June 2022 to May 2023, was compared to patients treated with cefoxitin from June 2023 to May 2024, following an institutional guideline update.

**Participants:**

Pregnant individuals aged 16 and older with diagnosed or presumed IAI were included.

**Results:**

Three hundred patients were included, 150 each in both the combination therapy and cefoxitin groups. Baseline characteristics were similar between the combination therapy and cefoxitin patients. Vaginal delivery was the most common mode of delivery, and rates of cesarean delivery were similar between groups. Most patients had a negative group B *Streptococcus* screen. Timely receipt of antimicrobials within 60–90 minutes of order entry occurred in significantly more patients in the cefoxitin group compared to combination therapy group (69.3% vs 4.7%, *P* < .001). Time to recommended therapy was significantly shorter in the cefoxitin group (76.4 ± 93.3 vs 183.7 ± 228.9 min, *P* < .001). There were no differences in mortality, 30-day infection-related readmission, or need for additional surgical/procedural intervention.

**Conclusions:**

Cefoxitin for IAI significantly improves timeliness of recommended antibiotic treatment, aligning with current guideline recommendations for prompt therapy. The absence of differences in secondary outcomes supports the clinical efficacy of cefoxitin and its consideration as a first-line agent in the management of IAI.

## Introduction

Treatment of intra-amniotic infection (IAI) has been shown to be variable among US obstetricians due to lack of data with modern antimicrobial treatment regimens to challenge long-standing traditional antimicrobial therapy recommendations.^1^ The American College of Obstetricians and Gynecologists (ACOG) defines IAI, or chorioamnionitis, as an infection that results in inflammation of the amniotic fluid, placenta, fetus, fetal membranes, or decidua. Due to the associated risks of neonatal morbidity and mortality, intrapartum antimicrobials are recommended when an infection is suspected or confirmed.^2^

ACOG IAI guidelines currently recommend combination therapy with ampicillin and gentamicin as the first-line treatment; clindamycin or vancomycin plus gentamicin is suggested for patients with severe penicillin allergies. Clindamycin or metronidazole is added to the recommended regimen of ampicillin and gentamicin at the time of umbilical cord clamping during a cesarean section. Alternative acceptable antimicrobial regimens listed in the ACOG guidelines include piperacillin–tazobactam, ampicillin–sulbactam, ertapenem, cefotetan, or cefoxitin with the recommendation to consider local antimicrobial data if selecting these alternatives. Following an institutional guideline update, cefoxitin, a broad-spectrum cephalosporin, is now utilized as first-line therapy for IAI beginning June 2023.^2^

The benefits of using cefoxitin as first-line therapy include its coverage against a wide range of gram-positive and gram-negative organisms, including anaerobes, making it a single-agent option for the treatment of IAI. This contrasts with the combination regimen of ampicillin, gentamicin, with or without clindamycin, which presents several concerns. Although generally well-tolerated, ampicillin has been suggested in animal studies to exacerbate placental inflammation in group B *Streptococcus* (GBS)-induced chorioamnionitis, potentially due to bacterial lysis.^3^ Gentamicin, although effective, is a known nephrotoxin and is associated with acute kidney injury (AKI).^4^ Another important consideration is that co-administration of ampicillin and gentamicin through a *Y*-site is not recommended due to potential compatibility issues. Clindamycin linked to an increased incidence of *Clostridioides difficile* infections, and national rising resistance rates to clindamycin in GBS are particularly concerning for its continued use in chorioamnionitis.^5^ It is recognized that *C. difficile* remains a concern with all antibiotics, including cefoxitin, though the risk is greatest with cumulative antimicrobial exposures.

Moreover, recent clindamycin drug shortages have highlighted the need for alternative regimens^6,7^ Few modern studies have directly compared cefoxitin with the first-line combination therapy regimen. Following the clindamycin shortage, institutional guidelines at our facility were updated for chorioamnionitis and endometritis, a review of which demonstrated that cefoxitin, when used as first-line therapy for chorioamnionitis, endometritis, or septic abortion, was noninferior to the standard antimicrobial regimen, underscoring its potential viability as a first-line treatment option.^8^

The goal of this follow-up study is to compare timeliness of antibiotic administration between cefoxitin and combination therapy (ampicillin and gentamicin), using administration within 60–90 minutes as the target for recommended antibiotic delivery. ACOG guidelines recommend the administration of antibiotics as soon as the diagnosis of chorioamnionitis is made.^2^ This recommendation aligns with the sepsis guidelines from the Society of Critical Care Medicine and the American College of Chest Physicians, which advocate for the expeditious administration of antibiotics—ideally within 1 hour—following recognition of sepsis or septic shock.^9^

## Materials and methods

This was a retrospective, observational before-and-after study conducted at a multi-site healthcare system with 8 obstetric hospitals. The first group, the combination therapy group, included patients treated with ampicillin and gentamicin, with or without clindamycin, between June 1, 2022, and May 31, 2023. The second group, the cefoxitin group, consisted of patients treated with cefoxitin monotherapy for suspected or diagnosed chorioamnionitis between June 1, 2023, and May 31, 2024. These groups were established to align with institutional guideline updates that designated cefoxitin as a first-line agent for IAI following a clindamycin shortage. The study received approval from the Prisma Health Institutional Review Board.

Eligible participants included pregnant individuals aged 16 years or older who received at least 1 dose of recommended antibiotics for suspected or diagnosed chorioamnionitis. Recommended antibiotics in this context include cefoxitin or the combination regimen. Individuals that received both cefoxitin and combination therapy or antibiotics for other indications (eg, GBS, surgical site infection [SSI] prophylaxis) were excluded from the study. Patients were identified through a data analytics tool integrated with the electronic health record (EHR), which flagged individuals with a diagnosis code for chorioamnionitis and corresponding antibiotic orders for either ampicillin or cefoxitin, depending on group assignment. Upon confirmation of study eligibility, chart review was performed to capture relevant clinical outcomes and demographic information.

Antibiotics for IAI are made available on obstetric units in automated dispensing cabinets due to lack of overnight coverage of pharmacy services at all included hospitals. Ampicillin and cefoxitin are both available at standard 2 g doses to be administered as intravenous (IV) piggyback over 30 minutes every 6 and 8 hours, respectively. Clindamycin, when used, is provided as a premixed 900 mg infusion over 30-minute infusion every 8 hours. Compounded doses of 80, 100, and 120 mg gentamicin are also available in automated dispensing cabinets to be administered as IV piggyback over 30 minutes each; patients who receive a dose of 160 or 200 mg would receive 2 bags infused sequentially over 60 minutes total. Automated EHR dosing algorithms round and verify gentamicin doses to determine whether 1 or 2 bags are dispensed, thereby minimizing bedside weight-based calculations, compounding time, and medication delivery delays. Some hospitals within the study site that have around-the-clock pharmacy services utilize patient-specific dosing, real-time medication preparation, and extended-interval dosing with gentamicin infusing over 60 minutes.

The primary outcome compared the number of patients who administered cefoxitin monotherapy to those who received combination therapy of ampicillin plus gentamicin 60 or 90 minutes. Timing was defined as the interval from the time the order is placed (time zero) to the completion of the antibiotic infusion(s), for either cefoxitin or both combination therapy agents. Cefoxitin and ampicillin were formulated to be infused over 30 minutes, so all patients in the cefoxitin group were analyzed using 60 minutes as the goal time to recommended antibiotics. In contrast, the time threshold for the combination therapy group varied based on gentamicin dosing requirements: patients requiring a single gentamicin bag were assessed using a 60-minute window, whereas those requiring 2 sequential bags or who utilized extended infusion were assessed using a 90-minute window to account for the additional infusion time. This variability reflects real-world operational constraints.

Secondary outcomes included several key comparisons and incidence measures. The study compared antibiotic administration times as a continuous variable. Clinical outcomes evaluated include incidence of infection-related readmissions within 30 days, such as endometritis and SSIs defined by the National Healthcare Safety Network, hospital length of stay, and hypersensitivity reactions that lead to discontinuation of the antibiotic prior to completion of the first dose or necessitated a change in the antibiotic regimen during treatment.^10^ Additional variables analyzed included the duration of antibiotic therapy and the frequency of serum creatinine monitoring for patients receiving gentamicin. The study also assessed the incidence of AKI, which was defined as an absolute rise in serum creatinine of 0.3 mg/dL or greater from the patient-specific baseline within 48 hours, or an increase of at least 1.5 times the baseline within 7 days.^11^ Serum creatinine and AKI were assessed only in patients receiving gentamicin, as monitoring in this group aligns with institutional policy and procedure for aminoglycoside dosing.

A power calculation determined that 140 patients were required in each group to achieve 80% power. This calculation was based on an estimation that 5% of patients in the combination therapy group and 15% in the postcefoxitin group would meet the primary end point. These expected rates were derived from sepsis literature, where prompt antibiotic administration is strongly recommended.^17^ Continuous variables were tested using Student’s *t* test or Wilcoxon rank sum, respectively. Discrete variables were tested using χ^2^ test or Fisher’s exact test for small sample sizes. Values of *P* < .05 were considered statistically significant. All analyses were carried out using R statistical software (R Foundation for Statistical Computing, version 4.0.2, Vienna, Austria).

## Results

A total of 300 patients were included in the study, with 150 patients in each group: the cefoxitin group and the combination therapy group. In the combination therapy group, 10 patients were excluded prior to reaching the 150-patient target due to not receiving gentamicin. In the cefoxitin group, 7 patients were excluded because they received ampicillin and/or gentamicin in addition to cefoxitin.

Patient demographics are summarized in Table 1. The combination therapy and cefoxitin groups were similar in terms of age, race, hospital length of stay, and intensive care unit admission rates. A higher proportion of patients in the cefoxitin group identified as Hispanic or Latino compared to the combination group (47 [31.3%] vs 31 [22.7%]).

**Table 1.** Patient demographics

	Combination therapy (n = 150)	Cefoxitin (n = 150)	*P*-value
Age in years, mean ± SD	25.4 ± 5.6	26.3 ± 6.0	.269
Race, n (%)			.174
American Indian/Alaska Native	0 (0)	0 (0)	
Asian	2 (1.3)	7 (4.7)	
Native Hawaiian or other	0 (0)	0 (0)	
Pacific Islander			
Black or African American	43 (28.7)	31 (20.7)	
White	68 (45.3)	61 (41.7)	
Hispanic	28 (18.7)	41 (27.3)	
More than one race	4 (2.7)	5 (3.3)	
Unknown/not reported	5 (3.3)	5 (3.3)	
Ethnicity, n (%)			.180
Hispanic or Latino	34 (22.7)	47 (31.3)	
Not Hispanic or Latino	115 (76.7)	102 (68)	
Unknown/not reported	1 (0.7)	1 (0.7)	
Hospital length of stay in days, median (IQR)	3.3 (2.8, 4.0)	3.2 (2.8, 4.0)	.454
ICU admission, n (%)	0 (0)	1 (0.7)	1.000
Gentamicin dosing, n (%)			
One-bag	77 (51.3)		
Two-bag	47 (31.3)		
Extended interval dosing	26 (17.3)		

n, number of patients; SD, standard deviation; IQR, interquartile range; ICU, intensive care unit.

Delivery characteristics are detailed in Table 2. No significant differences were observed between groups in terms of mode of delivery, labor induction, premature rupture of membranes (PROM), or preterm PROM. Among patients who underwent cesarean section, the proportion of those who had labored cesareans was similar between groups. Notably, although the difference was not statistically significant, a greater number of patients in the postcefoxitin group had an unknown GBS screening status (19 [12.7%] vs 8 [5.3%]).

**Table 2.** Delivery characteristics

	Combination therapy (n = 150)	Cefoxitin (n = 150)	*P*-value
Mode of delivery, n (%)			.172
Abortion	3 (2.0)	8 (5.3)	
Vaginal	95 (63.3)	83 (55.3)	
C-section	52 (34.7)	59 (39.3)	
Labored cesarean, n (%)	46 (30)	57 (38)	.246
Induction, n (%)	92 (62)	88 (58.7)	.724
Premature rupture of membranes, n (%)			1
PROM	5 (3.3)	5 (3.3)	.852
PPROM	17 (11.3)	15 (11.3)	1
Prolonged rupture of membranes	10 (6.7)	10 (6.7)	
None	119 (79.3)	120 (80)	1
Group B *Streptococcus*, n (%)			.084
Positive	24 (16)	21 (14)	
Negative	118 (78.7)	110 (73.3)	
Unknown	8 (5.3)	19 (12.7)	
Number of patients that received clindamycin, n (%)	57 (38)	4 (2.6)	

n, number of patients; PROM, premature rupture of membranes; PPROM, preterm premature rupture of membranes.

Clinical outcomes are summarized in Table 3. Significantly more patients in the cefoxitin group met the primary outcome of administration of antimicrobials within 60–90 minutes compared to the combination group (104 [69.3%] vs 7 [4.7%], *P* < .001). The time to recommended antibiotic administration was significantly shorter in the cefoxitin group compared to the combination group (76.4 ± 93.3 min vs 183.7 ± 228.3 min). No other secondary outcomes demonstrated statistically significant differences between the groups.

**Table 3.** Results

	Combination therapy (n = 150)	Cefoxitin (n = 150)	*P*-value
Receipt of antimicrobials within 60–90 min of order entry, n (%)	7 (4.7)	104 (69.3)	<.001
Duration of therapy, days ± SD	1.28 ± 1.32	1.25 ± 0.89	.837
Time to recommended therapy, min ± SD	183.7 ± 228.9	76.4 ± 93.3	<.001
Mortality, n (%)	0 (0)	0 (0)	1
Infection-related readmission within 30 d, n (%)	4 (2.7)	7 (4.7)	.541
Additional surgical/procedural interventions, n (%)	9 (6)	10 (6.7)	1
Patients on gentamicin for greater than 48 h, n (%)	7 (4.7)	–	–
Incidence of AKI on gentamicin for greater than 48 h, n (%)	7 (100)	–	–

n, number of patients; SD, standard deviation; AKI, acute kidney injury.

Infection-related readmission within 30 days: Any infection requiring admission to the hospital.

Incidence of AKI on gentamicin for greater than 48 hours: As defined by KDIGO guidelines.^11^

### Discussion

This retrospective cohort study of patients with suspected or confirmed chorioamnionitis at a multi-site health system demonstrated that patients were more likely to receive recommended antibiotics for IAI in an optimal time frame when cefoxitin was utilized compared to combination antibiotics. There were no differences observed in any of the additional secondary outcomes, including 30-day infection-related readmission, need for further surgical or procedural interventions, or overall mortality. This study highlights the advantages of cefoxitin in terms of timeliness of antibiotic therapy, compared to the current first-line regimen of ampicillin and gentamicin, in a setting where ACOG recommends ‘immediate’ administration of antibiotics.^2^

The current ACOG guidelines for the management of IAI recommend ampicillin and gentamicin as first-line agents, based on historical studies. These recommendations have not been revisited in recent years, despite growing concerns about the adverse effects associated with aminoglycosides, which are increasingly falling out of favor in clinical practice for non-pregnant patients, and improved safety profile for other antimicrobials such as cephalosporins like cefoxitin.^13^ Notably, the use of aminoglycosides in IAI remains one of the few areas where they are still recommended as part of the first-line therapy options. Historically, few studies have explored cefoxitin as a potential treatment for chorioamnionitis.

Several studies from the 1980s demonstrated comparable efficacy between cefoxitin and other antimicrobial regimens for obstetric and gynecologic infections.^14–17^ However, many of the comparator agents like moxalactam and mezlocillin are no longer available for therapeutic use, these studies were not specific for chorioamnionitis, and the data are outdated by newer literature. A 2024 study analyzed the use of cefoxitin for chorioamnionitis in the context of evolving institutional practices and recent clinical data.^8^ The review highlights a large retrospective cohort study in which a healthcare system transitioned to first-line cefoxitin monotherapy for IAIs, including chorioamnionitis. This change was associated with noninferior rates of serious maternal clinical events postdelivery compared to traditional regimens such as ampicillin plus gentamicin, after adjusting for baseline differences between groups. The study emphasizes the importance of local resistance patterns and institutional protocols in guiding antibiotic selection but supports the adoption of cefoxitin as an evidence-based treatment option for the management of chorioamnionitis.

ACOG identifies several single-agent alternatives to ampicillin plus gentamicin that vary in antimicrobial spectrum and operational practicality. Ampicillin–sulbactam and piperacillin–tazobactam provide broad coverage of the polymicrobial pathogens involved, including anaerobes; however, the expanded gram-negative coverage of piperacillin–tazobactam is generally unnecessary for routine chorioamnionitis and may raise stewardship concerns. Although recent literature compared combination therapy to piperacillin–tazobactam, including a retrospective study that demonstrated piperacillin–tazobactam was noninferior to ampicillin plus gentamicin for IAI, with no significant differences in composite maternal or neonatal morbidity after propensity-score matching.^18^ Ertapenem provides the most convenient once-daily dosing and broad-spectrum activity but may be limited by cost, antimicrobial stewardship considerations, and reduced reliability in critically ill or hypoalbuminemic patients. All single-agent regimens simplify care by eliminating the need for adjunctive clindamycin after cesarean delivery. Additionally, most of these alternatives can be administered as an IV push, improving timeliness and preserving IV access.

Cefoxitin, along with the other alternative regimens, may represent a more efficient and operationally streamlined alternative to ampicillin and gentamicin regimens for chorioamnionitis, offering both clinical effectiveness and workflow advantages. Many patients admitted to labor and delivery units often have only a single peripheral IV line available, which may complicate the administration of combination therapy and introduce potential for medication delays and errors. Additionally, gentamicin introduces further logistical complexity due to weight-based dosing requirements and the need for dose calculations that may depend on pharmacist availability, posing challenges in settings without 24-hour pharmacy support.

This study has several limitations that should be considered when interpreting the findings. First, the lack of standardized clinical documentation across patient records introduced variability, which may have led to inconsistencies in data interpretation and limited the ability to precisely identify the timing and rationale for clinical decisions. The primary outcome measure has not been validated and differs from Surviving Sepsis Campaign recommendations, which are based on time from sepsis recognition to antibiotic initiation. In this study, antibiotic order entry was used as “time zero,” and time to completion was measured, reflecting EHR limitations, as the exact time of clinical recognition is not reliably recorded. Thus, order time served as a pragmatic surrogate that may not fully capture the onset of clinical suspicion, and the 60- to 90-minute time frame was inferred.

Variability in gentamicin dosing protocols within the study site is another limitation which may limit comparisons across patients and healthcare settings. Importantly, the absence of neonatal outcome data limits the ability to comprehensively evaluate the overall efficacy of cefoxitin. Additionally, AKI was evaluated only among patients receiving gentamicin, given its known risk of nephrotoxicity, which limits our ability to directly compare this safety outcome with cefoxitin. Lastly, this study is subject to potential unmeasured confounding variables, including but not limited to factors such as staffing variability, patient census and workload, and delays or inconsistencies in clinical documentation.

The use of cefoxitin for the treatment of chorioamnionitis was associated with a reduced time to recommended antibiotic administration, demonstrating improved efficiency in initiating appropriate therapy. Although no significant differences were observed in secondary clinical outcomes, these findings contribute to the growing body of evidence supporting the efficacy and clinical utility of cefoxitin in the management of chorioamnionitis.

## Research transparency and reproducibility

The data that support the findings of this study are available from the corresponding author upon reasonable request.
